# PTSD in U.S. Veterans: The Role of Social Connectedness, Combat Experience and Discharge

**DOI:** 10.3390/healthcare6030102

**Published:** 2018-08-22

**Authors:** Sara Kintzle, Nicholas Barr, Gisele Corletto, Carl A. Castro

**Affiliations:** USC Suzanne Dworak-Peck School of Social Work, University of Southern California, 1150 S. Olive Street Suite 1406, Los Angeles, CA 90015, USA; nicholub@usc.edu (N.B.); gcorlett@usc.edu (G.C.); cacastro@usc.edu (C.A.C.)

**Keywords:** social connectedness, military, veterans, combat, discharge status, PTSD, mental health

## Abstract

Service members who transition out of the military often face substantial challenges during their transition to civilian life. Leaving military service requires establishing a new community as well as sense of connectedness to that community. Little is known about how social connectedness may be related to other prominent transition outcomes, particularly symptoms of posttraumatic stress disorder (PTSD). The purpose of this study was to explore the role of social connectedness in the development of PTSD, as well as its relationship to the known risk factors of combat exposure and discharge status. Data used were drawn from a needs assessment survey of 722 veterans. A path model was specified to test direct and indirect effects of combat experiences, non-honorable discharge status, and social connectedness on PTSD symptoms. Results demonstrated positive direct effects for combat experiences and non-honorable discharge status on PTSD symptoms while social connectedness demonstrated a negative direct effect. Both combat experiences and non-honorable discharge status demonstrated negative direct effects on social connectedness and indirect on PTSD through the social connectedness pathway. Study findings indicate social connectedness may be an important factor related to PTSD in veterans as well as an intervention point for mitigating risk related to combat exposure and discharge status.

## 1. Introduction

Serving in the U.S. military provides a substantial opportunity for a sense of community, belonging and understanding. Re-establishing a new sense of community is essential for veterans transitioning out of the military and into civilian life. However, developing a new sense of connectedness is one of many challenges separating service members may face. Little is known about the role social connectedness may play in promoting positive transition outcomes.

One such challenge transitioning veterans may encounter is unmet mental health needs, particularly posttraumatic stress disorder (PTSD). Evidence suggests that veterans are especially at risk of developing PTSD due to the potential stressors associated with combat exposure and military-related trauma. The prevalence of PTSD among veterans ranges from 11 to 30% based on the area of service [1,2]. Research indicates that an estimated 30% of Vietnam, 10% of Gulf War, 15% of Iraq veterans and 11% of veterans returning from Afghanistan struggle with PTSD [1,2,3]. PTSD is a result of traumatic or stressful life events, and it can interfere with social, physical and psychological functioning. PTSD is characterized by intrusive thoughts in which the trauma is re-experienced, avoidance of situations that might trigger the trauma, a state of hyperarousal or vigilance and negative alterations in cognition and mood which may cause irritability and aggression [4,5]. Veterans with trauma exposure and PTSD are more susceptible to sleep disorders, mood changes, reckless behavior, substance use and isolation which may impede a successful transition from military to civilian life [1,5].

One of the primary risk factors for the development of PTSD is combat exposure. Extensive research identifies combat exposure as a strong predictor of health and psychological complications in veterans due to the risk of physical injury, psychological trauma and other stressors related to war [6,7]. A recent study examined associations between combat exposure and physical and psychological health focusing on the physical pain, PTSD and depression in veterans. The findings indicate that veterans exposed to combat had greater pain intensity and as a result higher PTSD and depression symptoms in comparison veterans without combat exposure [7].

Another factor associated with negative outcomes during transition is non-honorable discharge status. As qualification for benefits is determined by discharge status, the roughly 16% of veterans who leave the military with non-honorable discharge status are often met with additional barriers to getting care, which can create significant transition challenges [8,9]. With limited access to services, veterans with non-honorable discharge status are at increased risk of adverse mental health outcomes including PTSD [10].

Among the challenges that military veterans encounter during their transition from military to civilian life is a loss of social connectedness. Social connectedness refers to an individual’s internal sense of belongingness to the social environment [11]. Social connectedness impacts interpersonal relationships, peer affiliation, memberships, social behavior and overall social integration throughout the lifespan [12]. There are health and psychological benefits linked to social connectedness. Documented benefits to social connectedness include intimacy, sense of sharing and belonging [13]. Existing research shows that experiencing a higher sense of social connectedness may serve as a protective factor against psychological distress, depression, PTSD, low self-esteem and suicidal ideation [14,15]. The need for social connection is fundamental to the successful reintegration of veterans into civilian life. With the loss a sense of community, identity and belongingness often provided by the military, the inability to find a new sense of social connectedness may create difficulty for veterans interacting in the civilian world. This can lead to isolation and further transition challenges.

While it is well established that veterans may struggle in their transition out of the military and into civilian communities, little has been done to examine this transition in relation to social connectedness and its impact on risk factors and transition outcomes. The purpose of this study is to explore the effect of combat exposure, non-honorable discharge status, and social connectedness on PTSD symptoms in individuals who have served in the U.S. military.

## 2. Materials and Methods

Data used for this study were drawn from a community-based needs assessment involving veterans living in the San Francisco Bay Area [16]. Multiple recruitment strategies were utilized to achieve representativeness of the veteran population in the San Francisco Bay Area. Utilizing two methods for reaching potential participants, researchers partnered with higher-learning institutions and local agencies which serve veterans in the San Francisco Bay Area. The first method utilized an online survey approach by which the agency would send out an invitation and survey link to veterans within their database. The second method used an on-the-ground survey approach by which agencies would work with researchers to organize data-collection events within their respective organizations. Those who agreed to participate were sent either a paper survey copy or the online survey link. The final sampling strategy used print advertisements and social media to reach out to veterans within the San Francisco community. Avenues such as Facebook, Twitter, LinkedIn, mass emails and the survey website promoted the survey opportunity to potential participants. The survey took approximately 30 to 90 min to complete. All participants received a $15 gift card. All data collection procedures were approved by the University of Southern California (UP-15-00697) Institutional Review Board.

### 2.1. Measures

PTSD: The Posttraumatic Stress Disorder Checklist 5 (PCL-5) [17], a 20-item measure on a 0–4 Likert-type scale, was used to measure PTSD. The PCL-5 is a four-factor measure indexing disturbances in intrusive thoughts, physical arousal, cognitive, and avoidance symptoms. Responses to the 20 items result in a score from 0–80, with a cut-point score of 33 [18]. Sample items include “in the past month, how much were you been bothered by: Repeated, disturbing, and unwanted memories of the stressful experience?” and “in the past month, how much were you been bothered by: Feeling jumpy or easily startled?” Cronbach’s alpha for the PCL was 0.97 in these data.

Combat experiences: The 13-item short version of the Combat Experiences Scale [19] was used to measure combat experiences. Scale items are dichotomous and capture common impactful deployment-related experiences like receiving enemy fire and seeing dead bodies. Scores range from 0–13. Cronbach’s alpha for the CES was 0.90 in these data.

Discharge status: Participants reported honorable discharge or one of seven possible non-honorable discharge statuses, including general under honorable conditions, other than honorable, bad conduct discharge, dishonorable discharge, dismissal (officer), uncharacterized or other. These categories were summed to produce a dichotomous variable capturing any non-honorable discharge.

Social connectedness: The Social Connectedness Scale [20], an 8-item measure scored on a 6-point Likert scale, indexed social connectedness. The scale was developed based on the theory of self-psychology and measures feelings of belongingness. The scale was validated in a study with 626 undergraduate students and was found to have good test stability over a 2-week period (0.96) and strong internal reliability (0.91). Sample items include “I feel disconnected from the world around me” and “even around people I know, I don’t feel that I really belong”. Scores range from 8–48. Cronbach’s alpha for the Social Connectedness Scale was 0.94 in these data.

### 2.2. Analyses

First, bivariate correlations were computed for study variables of interest including combat experiences, discharge status, social connectedness, and PTSD symptoms (See Table 1). Next, a path model was specified to test the direct and indirect effects of combat experiences, non-honorable discharge status, and social connectedness on PTSD symptoms. The estimator was maximum likelihood with missing data. Direct effects were modeled for each of the three predictors, and indirect effects were modeled for both combat experiences and non-honorable discharge status through the social connectedness pathway. Normality of residual distributions for continuous predictors combat experiences and social connectedness were tested using post-estimation residual plots. All analyses were conducted in Stata [21].

## 3. Results

The final sample consisted of 722 veterans who lived in the San Francisco Bay Area and completed the survey. Over half of the sample identified as white (54%) and married or with a domestic partner (51%). One-quarter of the sample was aged over 60 while 37% were between the ages of 30 and 39. Thirty percent of the sample indicated receiving a non-honorable discharge status. The sample as a whole reported high levels of PTSD symptoms (*M* = 36.07, *SD* = 21.6) and moderate levels of social connectedness (*M* = 32.15, *SD* = 13.27). Sample demographics are displayed in Table 2.

Combat experiences, social connectedness, and PTSD symptoms were significantly correlated at the bivariate level, as were non-honorable discharge status and PTSD symptoms. Despite the finding of a nonsignificant bivariate association between discharge status and social connectedness, theoretical and empirical considerations [22], including hypothesized indirect effects among study variables, warranted the testing of the full model.

**Figure 1:**
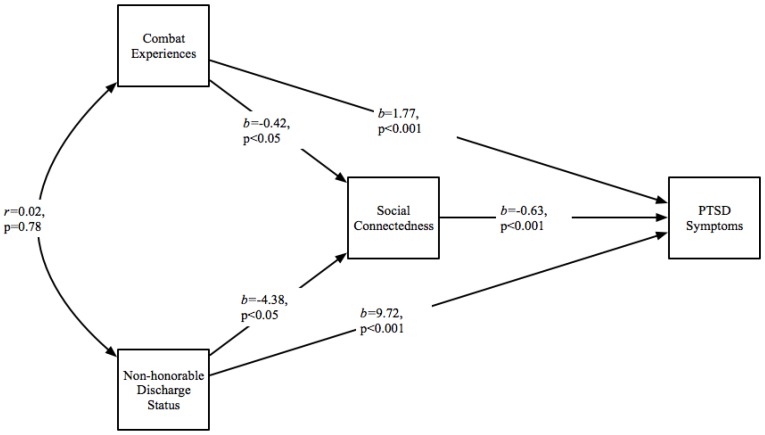
Path model with unstandardized direct effects.

In path analysis, the model was fully identified (*N* = 722). The final model with unstandardized direct effects is shown in Figure 1. Standardized direct, indirect, and total effects are reported below. As hypothesized, there were positive direct effects for combat experiences (β = 0.36, *p* < 0.001) and non-honorable discharge status (β = 0.21, *p* < 0.001) on PTSD symptoms. Social connectedness demonstrated a negative direct effect on PTSD symptoms (β = −0.41, *p* < 0.001). In addition, both combat experiences (β = −0.13, *p* < 0.05) and non-honorable discharge status (β = −0.14, *p* < 0.05) demonstrated negative direct effects on social connectedness. There were also significant indirect effects for both combat experiences (β = 0.05, *p* < 0.05) and non-honorable discharge status (β = 0.06, *p* < 0.05) on PTSD through the social connectedness pathway. The total effect for combat experiences on PTSD was β = 0.41, *p* < 0.001. The total effect for non-honorable discharge status on PTSD was β = 0.26, *p* < 0.001.

Figure 1 displays the fully identified (*N* = 722) path analysis model demonstrating a direct negative effects of social connectedness on PTSD symptoms and direct positive effects of non-honorable discharge status and combat experiences on PTSD symptoms. Also demonstrated are significant indirect effects for both combat experiences and non-honorable discharge status on PTSD through the social connectedness pathway.

## 4. Discussion

Study findings indicate social connectedness may be an important factor related to PTSD in veterans. Social connectedness had a direct effect on PTSD symptoms, indicating the extent to which veterans in the study felt socially connected inversely impacted the level of PTSD symptoms. As expected, both combat experience and a non-honorable discharge status had a positive direct effect on PTSD, indicating higher levels of combat experience and a non-honorable discharge status to be associated with more severe PTSD symptom severity. This aligns with the body of research which has examined the traumatic impact of combat as well as emerging literature on non-honorable discharge status as a risk factor for PTSD [5,10].

Combat experiences and non-honorable discharge status were also found to have an indirect effect on PTSD symptoms through their effects on social connectedness. This means that while these factors are present, they not only act on PTSD directly but also indirectly by inhibiting social connectedness. This indicates that veterans in the study who endorsed experiencing combat, as well as those who identified as having a non-honorable discharge status, were less likely to report feeling connected to individuals and their community. This, in turn, is associated with increased severity of PTSD symptoms. Conversely, social connectedness was negatively associated with PTSD and exerted the strongest direct effect of all predictors. This finding suggests that enhancing social connectedness may provide a robust buffering effect against the risks posed by combat experiences and non-honorable discharge status.

While there is limited research examining the role of social connectedness in the mental health of veterans, these findings are similar to those that have been found in other populations. Research has demonstrated social connectedness to have a significant positive impact on the physical and mental health of older adults [23,24]. Other studies have also found important connections between social connectedness and mental health in adolescents, transgender adults and college athletes [25,26,27].

Study findings have significant implications for considering PTSD in veterans. Although expected, both combat experiences and a non-honorable discharge status resulted in increased risk for PTSD. Identifying service members leaving the military with such risk factors should be identified early for intervention. This is particularly true for those with a non-honorable discharge status as these individuals may not have access to benefits that allow for mental health treatment. Results indicate social connectedness may be an important protective factor in the development of symptoms of PTSD. While researchers and practitioners often discuss the importance of reducing risk factors, combat exposure and discharge status are risk factors that occur during service. Increased social connectedness is a potential protective factor that can be targeted during and after the transition from service by assisting veterans in building a new community that provides a sense of belonging. While we know that such activities that target social connectedness are important to overall well-being, findings suggest they could be a significant contributor to positive mental health in veterans.

Further research is needed to examine social connectedness in veterans. The protective nature of social connectedness should be explored further in relation to the broad military transition experience. Early interventions during transition related to social connectedness could be an avenue for protecting veterans for negative outcomes related to suicide risk, mental and physical health. Further research should detail these relationships. It is also imperative that practitioners working with veterans take the time to explore feelings of social connectedness and encourage activities that promote a sense of connection to individuals and the community.

## 5. Conclusions

While transition from the military has received considerable attention as a point of intervention for promoting veteran success, little has been done to explore the role community and social connections may have in encouraging a positive transition. Findings demonstrate that facilitating the development of new social connections may serve as a protective factor in the development of PTSD symptoms. The results also indicate this may be particularly true for vulnerable veterans, such as those who have experienced combat and veterans with a non-honorable discharge status. 

## Figures and Tables

**Table 1:** Bivariate correlations for variables of interest.

Variable	Combat Experiences	Non-Honorable Discharge Status	Social Connectedness	PTSD Symptoms
Combat experiences	1			
Non-honorable discharge status	0.01	1		
Social connectedness	−0.14 *	−0.09	1	
PTSD symptoms	0.40 ***	0.26 ***	−0.40 ***	1

* *p* < 0.05, *** *p* < 0.001. Pearson’s product moment correlations were computed for continuous by continuous associations. Biserial correlations were computed for non-honorable discharge status and continuous variables.

**Table 2:** Sample descriptive statistics.

Variable	Variable Descriptors	%	*N* = 722	M	SD
**Age**	18–29	8.61%	62		
	30–39	37.36%	269		
	40–49	16.25%	117		
	50–59	12.91%	93		
	60 and older	15.69%	179		
	70 and older	9.17%	66		
**Gender**	Male	80.56%	580		
	Female	19.03%	137		
	Transgender	0.40%	3		
**Marital Status**	Single	28.02%	202		
	Married/Domestic Partnership	51.04%	368		
	Divorced/separated	17.89%	129		
	Widowed	3.05%	22		
**Race/Ethnicity**	American Indian/Alaska Native	2.78%	20		
	Asian	6.40%	46		
	Black	17.25%	124		
	Hawaiian/Pacific Islander	2.50%	18		
	White	54.38%	391		
	Hispanic/Latino	11.82%	85		
	Other	4.87%	35		
**Education**	Some High School	2.22%	16		
	GED	3.47%	25		
	High school diploma	9.29%	67		
	Some college	26.49%	191		
	Associate degree	12.48%	90		
	Bachelor’s	23.30%	168		
	Master’s	18.45%	133		
	Doctorate	2.50%	18		
	Other	1.80%	13		
**Non-Honorable Discharge Status**		29.64%	214		
**Combat Experiences**				6.64	4.38
**Social Connectedness**				32.15	13.27
**PTSD Symptoms**				36.07	21.6
